# Sonic hedgehog pathway for the treatment of inflammatory diseases: implications and opportunities for future research

**DOI:** 10.1136/jitc-2021-004397

**Published:** 2022-06-16

**Authors:** Marco Palla, Luigi Scarpato, Rossella Di Trolio, Paolo Antonio Ascierto

**Affiliations:** Melanoma, Cancer Immunotherapy and Innovative Therapy, Istituto Nazionale Tumori IRCCS Fondazione G. Pascale, Napoli, Italy

**Keywords:** COVID-19, Inflammation Mediators, Inflammation

## Abstract

The Sonic hedgehog (Shh) signaling pathway is an essential pathway in the human body that plays an important role in embryogenesis and tissue homeostasis. Aberrant activation of this pathway has been linked to the development of different diseases, ranging from cancer to immune dysregulation and infections.

Uncontrolled activation of the pathway through sporadic mutations or other mechanisms is associated with cancer development and progression in various malignancies, such as basal cell carcinoma, medulloblastoma, pancreatic cancer, breast cancer and small-cell lung carcinoma. Targeted inhibition of the pathway components has therefore emerged as an attractive and validated therapeutic strategy for the treatment of a wide range of cancers. Currently, two main components of the pathway, the smoothened receptor and the glioma-associated oncogene homolog transcriptional factors, have been investigated for the development of targeted drugs, leading to the marketing authorization of three smoothened receptor inhibitors for the treatment of basal cell carcinoma and acute myeloid leukemia.

The Shh pathway also seems to be involved in regulating the immune response, possibly playing a role in immune system evasions by tumors, development of autoimmune diseases, such as rheumatoid arthritis and Crohn’s disease, airway inflammation, and diseases related to aberrant activation of T-helper 2 cellular response, such as allergy, atopic dermatitis, and asthma.

Finally, the Shh pathway is involved in pathogen-mediated infection, including influenza-A and, more recently, SARS-CoV-2 viruses. Therefore, agents that inhibit the Shh signaling pathway might be used to treat pathogenic infections, shifting the therapeutic approach from strain-specific treatments to host-based strategies that target highly conserved host targets.

## Introduction

The Sonic hedgehog (Shh) signaling pathway plays a key role in regulating the growth, differentiation and proliferation of several cells and tissues.^1 2^ In human pathology, it is widely recognized that Shh signaling is involved in the development of several types of cancer,^3–8^ including basal cell carcinoma,^7 8^ malignant gliomas, medulloblastoma, leukemias, and cancers of the breast, lung, pancreas, and prostate. Tumor genesis, progression and therapeutic response have all been impacted by the Shh signaling pathway.

The Shh pathway is highly complex; it involves multiple effectors and regulators and experiences central compartmentalized coordination in the primary cilium.^9^ The pathway is initiated by binding of Shh to the cell surface receptor Patched; this triggers the phosphorylation of the 7-transmembrane G protein-coupled receptor smoothened (SMO), which then enters the primary cilium to trigger the downstream events. The accumulation and activation of SMO leads to the translocation of suppressor of fused to the primary cilium, allowing its dissociation from the glioma-associated oncogene (GLI) family transcription factors. The GLI transcription factors (GLI1, GLI2 and GLI3) thus translocate into the nucleus, where they induce the expression of target genes and promote cell growth, survival and differentiation.^10 11^ In addition to this standard pathway, non-canonical SMO-independent signaling has been described in some tissues and cells.^12^

Both SMO and the GLI family of zinc finger transcription factors are regarded as important targets for cancer therapy. To date, two SMO receptor inhibitors (sonidegib and vismodegib) have received Food and Drug Administration (FDA) and European Medicines Agency (EMA) approval for treating basal cell carcinoma^13^ and another (glasdegib) has been recently approved by FDA for treating acute myeloid leukemia, while many clinical trials are being conducted to evaluate the efficacy of this exciting class of targeted therapy in a variety of other cancers.^4–6^

While most efforts have been devoted to pharmacologically targeting SMO, developing the GLI-targeted approach also has its merit. GLI proteins can be activated by both Shh ligand-dependent and Shh ligand-independent mechanisms. Moreover, it has recently been recognized that partial inhibition of multiple targets is more productive than a single hit, as it may reduce the chances of developing drug resistance.^13^ Agents that target GLI could be used to overcome the emergence of drug resistance to SMO, which is caused by different mechanisms, such as genetic mutations of SMO and other signaling molecules in the canonical Shh pathway, activation of the non-canonical Shh pathway, and loss of primary cilia.^14^ To date, a number of small molecules targeting GLI have been discovered and investigated, showing promising results in blocking tumor cell proliferation.^15^

The development of agents targeting other components of the pathway has proven challenging so far. For example, the upstream protein Shh, which is overexpressed in many cancers, has been considered a promising antitumor target, but only a few small molecules have been identified that can disrupt the Shh-Patched 1 protein (Shh-PTCH) interaction.^14 16 17^

Evidence has recently emerged that Shh signaling is also involved in the inflammatory and immune responses of different diseases. Some pathogens use this pathway to control the local infected environment. Among the human inflammatory diseases where Shh pathway is considered to play a role are an influenza-A infection, cigarette-induced airway inflammation, arthritis, inflammatory bowel diseases, pancreatitis, colitis-associated cancer, asthma, allergy, atopic dermatitis and T-cell polarization.^1 2 18–20^

This commentary will examine the current knowledge on the pathophysiological interactions of Shh signaling that make this pathway a potential target for future treatments in a wide range of human diseases besides various cancers.

## Hedgehog pathway and cancer diseases

The Shh signaling pathway plays an important role in normal embryonic tissue development by modulating the epithelial–mesenchymal interactions, which regulate cell proliferation and differentiation.

Aberrant activation of the Shh/GLI signaling pathway by various mechanisms may be responsible for the development of various human cancers. Targeting Shh/GLI remains a promising treatment strategy with the potential for curative effects by eradicating cancer stem cells involved in tumor initiation, metastasis and drug resistance. Upregulation of the Shh ligand in autocrine or paracrine fashion has been reported in pancreatic, gastrointestinal, breast, lung, brain and prostate cancers.^3–6^ Mutations of signaling pathway components are drivers of the basal cell nevus syndrome (Gorlin syndrome), leading to the development of skin basal cell carcinomas^7 8^ and risk of rhabdomyosarcoma and medulloblastoma. Shh-activating mechanisms also seem implicated in the pathogenesis of lymphoma, multiple myeloma, and chronic myeloid leukemia.^3–6^

**Figure 1:**
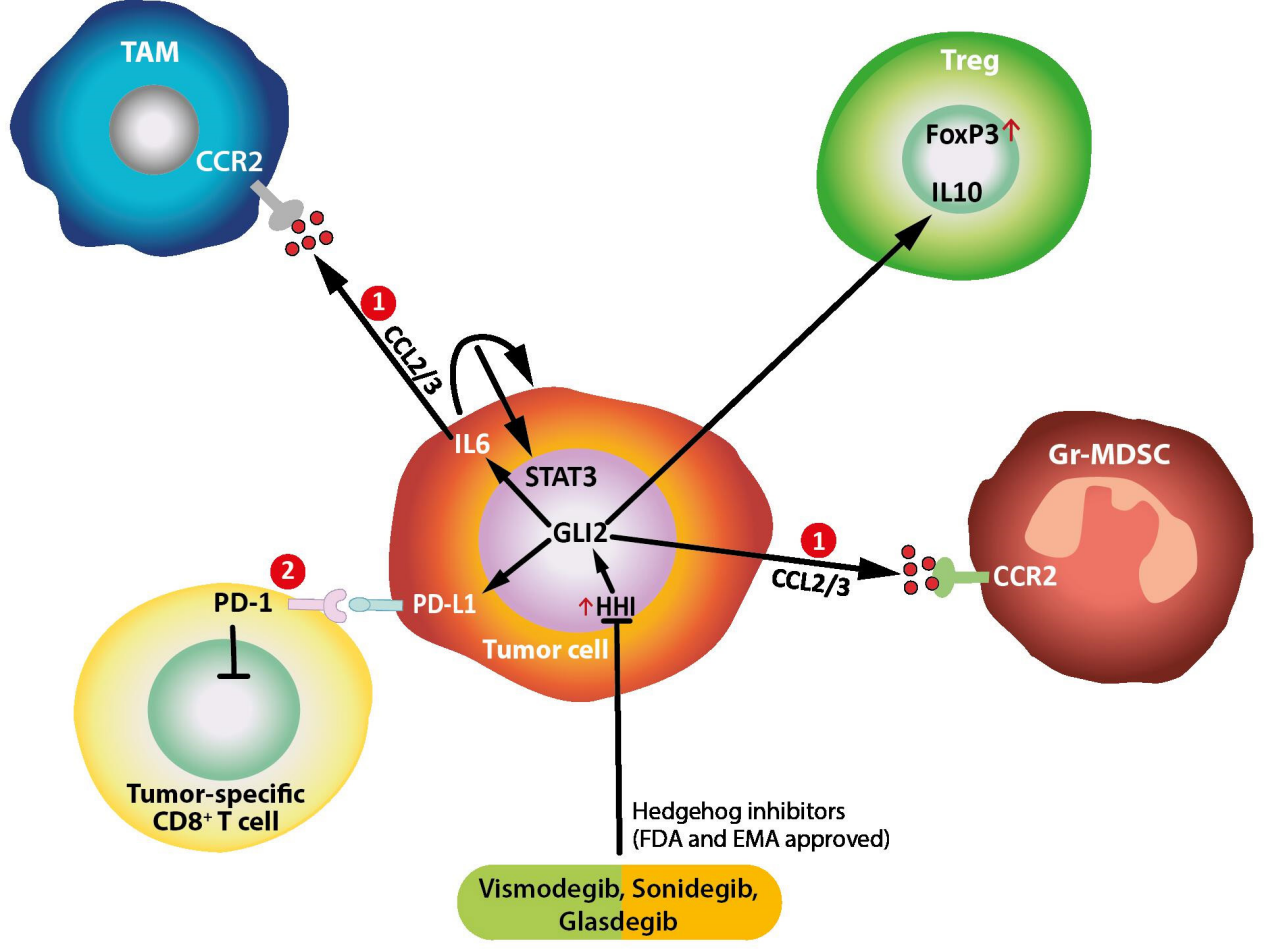
Mehanisms of immune modulation by HH/GLI signaling in cancer and inflammation. Cancer cell release CCL2/3 in response to oncogenic HH/GLI signaling, thereby recruiting TAMs and immunosuppressive MDSCs, 2. HH/GLI-induced PDL-1 expression in cancer and dendritic cells inhibits tumor specific cytotoxic T-cells via binding to PD-1. GLI, glioma-associated oncogene; HH, Hedgehog; MDSCs, myeloid-derived suppressor cells; TAMs, tumor-associated macrophages; FDA, Food and Drug Administration; EMA, European Medicines Agency.

The Shh/GLI signaling modulates inflammation in cancer by several mechanisms, as summarized in box 1 and depicted in figure 1.

Box 1 **Mechanisms of modulation of inflammation in cancer by the Sonic hedgehog/glioma (Shh/GLI) signaling pathways^4 7 8^**

- Cancer cells release CCL2/3 in response to oncogenic Shh/GLI signallingsignaling, thereby recruiting tumor-associated macrophages (TAMs) and immunosuppressive myeloid-derived suppressor cells.
- Shh/GLI-induced PD-L1 expression in cancer and dendritic cells inhibits tumor specific cytotoxic T-cells via binding to PD-1.
- GLI2 drives production of immunosuppressive cytokines and growth factors (IL-10 and transforming growth factor-β), which results in the inactivation of tumor-specific CD8 +T cells.
- Shh/GLI-induced IL-10 from stromal cells promotes FoxP3 expression in regulatory T-cells.
- Proinflammatory signals such as IL-6/signal transducer and activator of transcription 3 (STAT3) interact with Shh/GLI signallingsignaling; Shh/GLI-induced autocrine IL-6 signallingsignaling and/or proinflammatory IL-6 from TAM and stromal cells activate STAT3 signallingsignaling in cancer cells, thereby promoting malignant growth (Figure 1).
- In basal-cell carcinoma, the interaction of Shh/GLI and proinflammatory IL-6/STAT3 signaling synergistically regulates common GLI-STAT3 target genes and promotes cancer proliferation.

On the one hand, the activation of Shh/GLI generates an immune-suppressive response through the release of immune-suppressive molecules or by activating specific anti-inflammatory pathways in cancer and immune cells. Shh/GLI induces, for example, the expression of CCL-2/3 on cancer cells, thus recruiting tumor-associated macrophages (TAMs) and immunosuppressive myeloid-derived suppressor cells to the cancer lesion. Moreover, it stimulates cancer cells and dendritic cells to produce PD-L1, which inhibits tumor-specific cytotoxic T cells (CD8+) via the PD-1 receptor. Furthermore, the release of immune-suppressive cytokines and growth factors, such as interleukin-(IL)-10 and transforming growth factor-beta reduces the number of effector lymphocytes in the tumor tissue and enhance the polarization of T cells towards the formation of regulatory T-cell (Treg) formation and an anti-inflammatory environment.^4^

On the other hand, Shh/GLI can stimulate proinflammatory signals, which have been recognized as a potent promoter and enabler of malignant development when the activation of the immune system is persistent and inappropriate. Several studies have shown that during malignant development, Shh/GLI signaling and proinflammatory pathways, such as IL-6/signal transducer and activator of transcription-3 (STAT3) have reciprocal regulatory interactions, including tumor-promoting synergistic signal integration processes.^4^

Shh/GLI-induced autocrine IL-6 signaling and/or proinflammatory IL-6 from TAM and stromal cells activate STAT3 signaling in cancer cells, thereby promoting malignant growth (figure 1).

Moreover, in basal cell carcinoma, the interaction of Shh/GLI and proinflammatory IL-6/STAT3 signaling synergistically regulate common *GLI*/*STAT3* target genes and promotes cancer proliferation.^7 8^

The importance of the Shh/GLI signaling in oncogenic development and immune cross-talk and modulation makes this pathway a promising target for new anticancer therapeutic strategies.

In a rat model of colitis-associated cancer and human colon cancer cells, Shh inhibitors suppressed TNF-α-induced inflammatory signaling, especially IL-6/IL-6R/gp130 pathway.^21^ In a mouse model of colitis-associated cancer, Shh inhibitors significantly reduced tumor incidence and multiplicity, decreased the expression of IL-6, TNF-α, COX-2, STAT3, and NF-κB, and significantly induced apoptosis.^21^ Taken together, these data suggest that administration of Shh inhibitors could be an effective strategy to prevent colitis-induced colorectal carcinogenesis, mainly by targeting IL-6 signaling and suppressing oncogenic inflammation.

The signaling cascade identifies the transcription factor GLI1 as a central mediator of the IL-6 signaling network initiated in the tumor microenvironment, regulating the progression of pancreatic precursor lesions and tumor formation. When GLI1 is absent, IL-6 signaling from tumor-associated fibroblasts diminishes, and pancreatic precursor lesions do not progress to advanced stages.^22^

Inhibition of Shh signaling pathway with small molecules targeting the smoothened (SMO) can successfully inhibit the growth of various tumors.^23 24^ Saridegib, in combination with chemotherapy, contributed to suppressing serous ovarian cancer growth.^25^ Vismodegib, either alone or combined with standard chemotherapy agents, was effective in primary colorectal cancer xenografts.^26^ Vismodegib and sonidegib are currently FDA-approved drugs for patients with locally advanced and metastatic basal cell carcinoma.^13^ Glasdegib, alone or in combination with chemotherapy, showed antitumor activity in acute myeloid leukemia, myelodysplastic syndrome, and myelofibrosis,^27 28^ and has been recently approved by FDA as the first SMO receptor inhibitor for acute myeloid leukemia.^29^

Despite the promising results obtained by Shh pathway inhibitors, some challenges and concerns have emerged regarding the use of Shh inhibitors as immune modulators; given the complex and opposite effects, this pathway may have in terms of proinflammatory and anti-inflammatory signals. Studies in mice showed the importance of the anti-inflammatory cytokine IL-10 and Treg expression, mediated by Shh/GLI, in dampening the inflammation and preventing inflammatory damage in pancreatitis and colitis.^21 30^ Indeed, inhibition of Shh/GLI signaling worsened the progression of the inflammatory disease and promoted colitis-associated cancer development.^21^ In another study, de la Roche *et al* discovered that SMO is involved in T-cell activation and that administration of SMO inhibitors results in functional disruption of the immunological synapse and consequently loss of T-cell effector activity.^31^ The fact that SMO inhibitors might block the activity of T-cytotoxic cells could explain the failure of colon cancer trials, where drug targeting of Shh signaling accelerated cancer progression, forcing the termination of the clinical studies.^4^

Given the complexity and diverse effects of the Shh/GLI pathway on the immune microenvironment of malignant and non-malignant tissue, it is crucial to understand how deregulation of this signaling axis precisely alters antitumor immunity and tumor-promoting inflammation in order to support the development of more sophisticated tumor therapies.

## Hedgehog pathway and inflammation in viral pandemics

**Figure 2:**
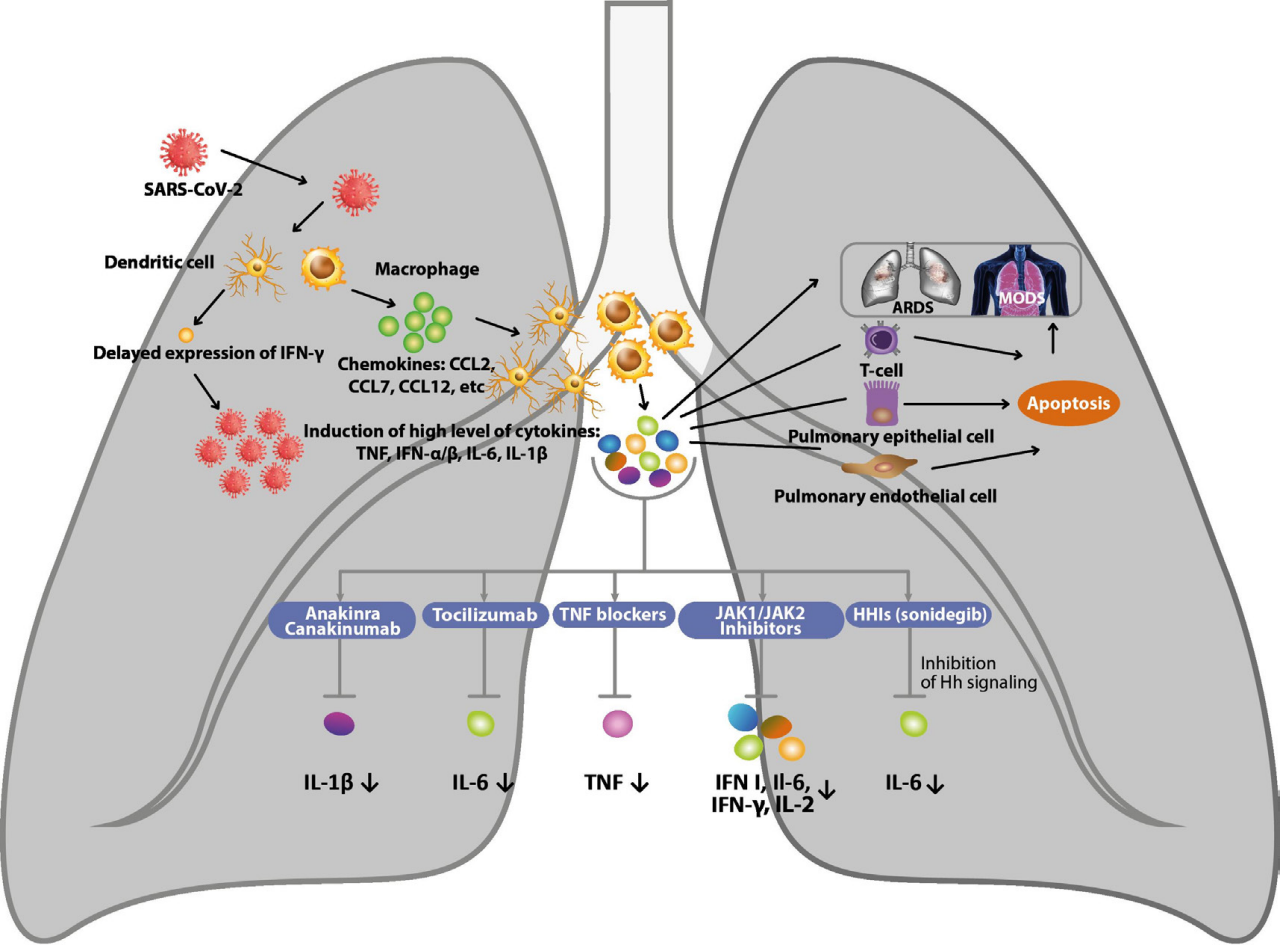
MechaTablenism of inhibiting the production of cytokine storm in COVID-19 and potential therapy. HH, hedgehog.

A study on infected mouse lungs and human lung cells transfected with NS1, a protein encoded by influenza-A virus, revealed an upregulation of Shh target genes, including IL-6.^32^ Since IL-6 levels were higher in animals infected with the more pathogenic virus carrying the A122V point mutation in *NS1*, the authors speculated that the hastened lethality caused by the mutant virus might be due, in part, to a Shh-dependent over-production of cytokines (the so-called cytokine storm), which have been considered the cause of past influenza pandemics (figure 2).^33 34^

A cytokine storm is an umbrella term encompassing several immune dysregulation disorders characterized by general symptoms and multiorgan dysfunction. The production of amounts of cytokines in excess to those required for a beneficial normal response is a life-threatening event that may result from the immune response to the pathogen with multiorgan dysfunction not directly related to the pathogen itself. Although laboratory abnormalities may be widely variable in cytokine storms, the increase in circulating cytokines is always present and associated with symptoms of acute systemic inflammation involving different organs. Excess of IL-6 may cause an impairment of natural killer-cell function leading to sustained activation of T cells, which may trigger the excessive production of several cytokines, such as interferon (IFN)-γ, IL-1, IL-6, TNF-α, and IL-18. The beneficial effects of specific monoclonal antibodies in reducing symptoms and improving organ functions have confirmed the key role of excessive cytokine production.^35^

The cytokine storm is considered the main mechanism underlying the devastating effects of SARS-CoV-2 pandemic named COVID-19.^33 34^ Serum cytokine levels found to be elevated in patients with severe COVID-19 include IL-1β, IL-6, IL-10, TNF-α, IFN-γ, macrophage inflammatory protein-1α and 1β, and VEGF. In particular, higher IL-6 levels are strongly associated with shorter survival. In addition to increased cytokine levels, circulating activated CD4 +and CD8+T cells and plasmablasts increase the spread of COVID-19. These changes are associated with laboratory abnormalities typical of cytokine storms, such as elevated C reactive protein and D-dimer levels, hypoalbuminemia, and renal dysfunction. Clotting abnormalities may be present in any disorder associated with cytokine storm, but thromboembolic events appear to be more frequent in COVID-19 than in other conditions.

One of the earliest therapies targeting cytokine storm in COVID-19 patients was the anti-IL-6 receptor monoclonal antibody tocilizumab.^36^ Despite the limited knowledge regarding the role of immune dysregulation and cytokine storm in COVID-19, several immunomodulatory drugs used for other cytokine storm disorders are currently under investigation.^37^ The anti-IL-1β monoclonal antibody canakinumab and the recombinant IL-1 receptor blocker anakinra have been used in patients with COVID-19 pneumonia and acute hypoxemic respiratory failure.^38 39^ Acalabrutinib, a selective inhibitor of Bruton tyrosine kinase regulating B-cell and macrophage signaling and activation, has also been proposed for dampening the hyperinflammatory response in COVID-19. Inhibitors of JAK1 and JAK2 approved for the treatment of a number of autoimmune and neoplastic conditions may have the potential to inhibit type-I IFN, IL-6, IFN-γ, and IL-2 signaling. However, anti-IL-6, inhibitors of Bruton tyrosine kinase and JAK could be unhelpful or even detrimental if given in the early phase of the disease, when the immune response to SARS-CoV-2 is adequate for controlling viral replication and clearance. Several viruses (influenza, Epstein-Barr, hepatitis B and C, HIV) can damage the host tissue by activating the Shh signaling pathway, for example, activation of STAT3 and upregulation of IL-6 expression, eventually driving to detrimental outcomes, such as fibrosis. Thus, it is conceivable that molecules inhibiting Shh signaling, such as sonidegib, have the potential for use as effective, broad-spectrum inhibitors of pathogenic responses to SARS-CoV-2 (box 2).

Box 2 **Several anti-inflammatory activities of hedgehog/glioma (HH/GLI) signaling**

**Tumor immunity^4^**

- Regulates immunosuppressive mechanisms, such as enhanced regulatory T-cell formation and production of immunosuppressive cytokines.
- In several cancer entities, aberrantly activated HH/GLI signaling drives tumor proliferation and growth, while simultaneously dampening inflammation and favors immunosuppression.

**Inflammation in basal cell carcinoma (BCC)^7^**

- IL-6 synergizes with HH/GLI in oncogenic transformation.
- The interaction of HH/GLI and proinflammatory IL-6/signal transducer and activator of transcription-3 (STAT3) signaling synergistically regulates common GLI–STAT3 target genes and promotes cancer promotion.

**Inflammation in colitis-associated cancer^21^**

- Intestinal epithelial cells TNF-α stimulated IEC-6 cells exhibit increased levels of proinflammatory cytokines, whereas SHH inhibitors suppress TNF-α-induced inflammatory signaling, especially IL-6/IL-6R/gp130 pathway.
- SHH inhibitors significantly reduce tumor incidence and multiplicity, decrease the expression of IL-6, TNF-α, COX-2, STAT3 and NF-κB, and significantly induce apoptosis.

**Autoimmune diseases^41 42^**

- Reduce paw swelling, serum levels of TNF-α, IL-1β, IL-6 and histological scores of joint damage in rats.
- Promotes cartilage extracellular matrix production with a potential clinical interest in rheumatoid arthritis treatment.

**Airway inflammation^43^**

- The HH pathway inhibitor cyclopamine attenuates the nicotine-induced increase in IL-6, IL-8 and TNF-α, while IL-10 levels increase, suggesting that the HH signaling pathway may partly contribute to cigarette-induced airway inflammation through the regulation of inflammatory mediators.
- Plays a key role in cigarette-induced airway inflammation, via the regulation of inflammatory mediators.

## Hedgehog pathway in other conditions

The hedgehog pathway seems to be involved in various diseases such as autoimmune diseases and airway inflammation.

Different components of the Shh pathway are expressed in the thymus and seem to be involved in multiple stages of T-cell development. In fact, Shh signaling influences the differentiation and proliferation of early thymocyte progenitors and plays a role in repertoire selection, clonal deletion of autoreactive cells and T-cell peripheral activation. Moreover, hedgehog proteins modulate T-cell receptor signaling, influencing T-cell differentiation into T helper 1 (T_H_1) or T helper 2 (T_H_2) or inducing cellular anergy.^40^ Recent in vitro evidence has shown that the Shh pathway promotes in particular T_H_2 differentiation of human CD4 +cells, thus suggesting that Shh signaling may be involved in the etiology of diseases mainly driven by this response, such as allergy, atopic dermatitis and asthma.^19^

Some studies also suggest the possible involvement of Hh signaling in autoimmune disorders such as rheumatoid arthritis and Crohn’s disease. Cyclopamine, a Hh inhibitor, attenuated inflammation and cartilage damage of adjuvant‐induced arthritis rats, as evidenced by reduced paw swelling, serum levels of TNF-α, IL-1β, IL-6 and histological scores of joint damage. GLI1 mRNA levels correlated negatively with type-II collagen and aggrecan mRNA levels, suggesting Hh signal inhibition was associated with promoting cartilage extracellular matrix production. These data suggest that inhibition of Hh signaling pathway might be of potential clinical interest in rheumatoid arthritis treatment.^41^

In Crohn’s disease, Hh signaling provides negative feedback to the lamina propria, down-regulating inflammatory cytokines and inhibiting leukocyte migration and fibroblast proliferation while favoring fibroblast migration. Therefore, Hh signaling appears to be strongly implicated in the pathogenesis of intestinal inflammation, thus representing a novel therapeutic target in inflammatory bowel diseases.^42^

Finally, in a study of human alveolar epithelial cells,^43^ the Hh pathway inhibitor cyclopamine attenuated the nicotine-induced increase in IL-6, IL-8 and TNF-α while IL-10 increased, suggesting that Hh signaling pathway may partly contribute to cigarette-induced airway inflammation via the regulation of inflammatory mediators. Thus, blocking Hh signaling and diminishing the airway inflammation reaction could be a potential approach to COPD therapy requiring investigation.

## Conclusion and future perspectives

Dysregulation of Shh/GLI signaling plays fundamental yet distinct roles in cancer and various chronic inflammatory diseases by exerting complex and diverse effects on the immune microenvironment of malignant and non-malignant tissues. Understanding the molecular rationale of how deregulation of the Shh/GLI signaling axis precisely alters immunity and inflammation will support the development of more sophisticated therapies. Shh signaling has also been shown to be a target for some pathogens that presumably use the pathway to control the local infected environment, thus justifying therapeutic uses of approved molecules that inhibit Hh signaling be expanded to treat pathogenic infections. In contrast to the currently available therapies, such as vaccines and antivirals, which target strain-specific and rapidly mutating viral proteins, treatments that target highly conserved host targets may ultimately provide superior and continual protection across a broader spectrum of strains. The emerging knowledge on the broad effects of the Shh signaling pathway may represent a rationale for their evaluation as a potential treatment of COVID-19.

## References

[1] Smelkinson MG. The hedgehog signaling pathway emerges as a pathogenic target. J Dev Biol 2017;5:14. 10.3390/jdb5040014 29214147 PMC5713906

[2] Rimkus TK, Carpenter RL, Qasem S, et al. Targeting the sonic hedgehog signaling pathway: review of smoothened and Gli inhibitors. Cancers 2016;8:22. 10.3390/cancers8020022 PMC4773745 26891329

[3] Carpenter RL, Ray H. Efficacy and safety of sonic hedgehog pathway inhibitors in cancer. Drug Saf 2019;42:263–79. 10.1007/s40264-018-0777-5 30649745 PMC6434684

[4] Grund-Gröschke S, Stockmaier G, Aberger F. Hedgehog/GLI signaling in tumor immunity - new therapeutic opportunities and clinical implications. Cell Commun Signal 2019;17:172. 10.1186/s12964-019-0459-7 31878932 PMC6933925

[5] Skoda AM, Simovic D, Karin V, et al. The role of the hedgehog signaling pathway in cancer: a comprehensive review. Bosn J Basic Med Sci 2018;18:8–20. 10.17305/bjbms.2018.2756 29274272 PMC5826678

[6] Girardi D, Barrichello A, Fernandes G, et al. Targeting the hedgehog pathway in cancer: current evidence and future perspectives. Cells 2019;8:153. 10.3390/cells8020153 PMC6406365 30759860

[7] Sternberg C, Gruber W, Eberl M, et al. Synergistic cross-talk of hedgehog and interleukin-6 signaling drives growth of basal cell carcinoma. Int J Cancer 2018;143:2943–54. 10.1002/ijc.31724 29987839 PMC6282712

[8] Leavitt E, Lask G, Martin S. Sonic hedgehog pathway inhibition in the treatment of advanced basal cell carcinoma. Curr Treat Options Oncol 2019;20:84. 10.1007/s11864-019-0683-9 31773379

[9] Bangs F, Anderson KV. Primary cilia and mammalian hedgehog signaling. Cold Spring Harb Perspect Biol 2017;9:a028175. 10.1101/cshperspect.a028175 27881449 PMC5411695

[10] Briscoe J, Thérond PP. The mechanisms of Hedgehog signalling and its roles in development and disease. Nat Rev Mol Cell Biol 2013;14:416–29. 10.1038/nrm3598 23719536

[11] Petrova R, Joyner AL. Roles for hedgehog signaling in adult organ homeostasis and repair. Development 2014;141:3445–57. 10.1242/dev.083691 25183867 PMC4197719

[12] Lee RTH, Zhao Z, Ingham PW. Hedgehog signalling. Development 2016;143:367–72. 10.1242/dev.120154 26839340

[13] Dummer R, Ascierto PA, Basset-Seguin N, et al. Sonidegib and vismodegib in the treatment of patients with locally advanced basal cell carcinoma: a joint expert opinion. J Eur Acad Dermatol Venereol 2020;34:1944–56. 10.1111/jdv.16230 31990414

[14] Stanton BZ, Peng LF, Maloof N, et al. A small molecule that binds hedgehog and blocks its signaling in human cells. Nat Chem Biol 2009;5:154–6. 10.1038/nchembio.142 19151731 PMC2770933

[15] Nguyen NM, Cho J. Hedgehog pathway inhibitors as targeted cancer therapy and strategies to overcome drug resistance. Int J Mol Sci 2022;23. 10.3390/ijms23031733. [Epub ahead of print: 03 Feb 2022]. PMC8835893 35163655

[16] Owens AE, de Paola I, Hansen WA, et al. Design and evolution of a macrocyclic peptide inhibitor of the sonic Hedgehog/Patched interaction. J Am Chem Soc 2017;139:12559–68. 10.1021/jacs.7b06087 28759213 PMC5753398

[17] Yun T, Wang J, Yang J, et al. Discovery of small molecule inhibitors targeting the sonic hedgehog. Front Chem 2020;8:498. 10.3389/fchem.2020.00498 32612978 PMC7309560

[18] Yánez DC, Papaioannou E, Chawda MM, et al. Systemic pharmacological smoothened inhibition reduces lung T-cell infiltration and ameliorates Th2 inflammation in a mouse model of allergic airway disease. Front Immunol 2021;12:737245. 10.3389/fimmu.2021.737245 34580585 PMC8463265

[19] Yánez DC, Lau C-I, Chawda MM, et al. Hedgehog signaling promotes T_H_2 differentiation in naive human CD4 T cells. J Allergy Clin Immunol 2019;144:1419–23. 10.1016/j.jaci.2019.07.011 31351102 PMC6843897

[20] Lau C-I, Yánez DC, Papaioannou E, et al. Sonic hedgehog signalling in the regulation of barrier tissue homeostasis and inflammation. Febs J 2021. 10.1111/febs.16222. [Epub ahead of print: 06 Oct 2021]. 34614300

[21] Kangwan N, Kim Y-J, Han YM, et al. Sonic hedgehog inhibitors prevent colitis-associated cancer via orchestrated mechanisms of IL-6/gp130 inhibition, 15-PGDH induction, Bcl-2 abrogation, and tumorsphere inhibition. Oncotarget 2016;7:7667–82. 10.18632/oncotarget.6765 26716648 PMC4884946

[22] Mills LD, Zhang Y, Marler RJ, et al. Loss of the transcription factor GLI1 identifies a signaling network in the tumor microenvironment mediating KRAS oncogene-induced transformation. J Biol Chem 2013;288:11786–94. 10.1074/jbc.M112.438846 23482563 PMC3636867

[23] Pietrobono S, Stecca B. Targeting the oncoprotein smoothened by small molecules: focus on novel acylguanidine derivatives as potent smoothened inhibitors. Cells 2018;7. 10.3390/cells7120272. [Epub ahead of print: 14 12 2018]. PMC6316656 30558232

[24] Xie H, Paradise BD, Ma WW, et al. Recent advances in the clinical targeting of Hedgehog/GLI signaling in cancer. Cells 2019;8:394. 10.3390/cells8050394 PMC6562674 31035664

[25] McCann CK, Growdon WB, Kulkarni-Datar K, et al. Inhibition of Hedgehog signaling antagonizes serous ovarian cancer growth in a primary xenograft model. PLoS One 2011;6:e28077. 10.1371/journal.pone.0028077 22140510 PMC3226669

[26] Yauch RL, Gould SE, Scales SJ, et al. A paracrine requirement for hedgehog signalling in cancer. Nature 2008;455:406–10. 10.1038/nature07275 18754008

[27] Savona MR, Pollyea DA, Stock W, et al. Phase Ib study of Glasdegib, a hedgehog pathway inhibitor, in combination with standard chemotherapy in patients with AML or high-risk MDS. Clin Cancer Res 2018;24:2294–303. 10.1158/1078-0432.CCR-17-2824 29463550

[28] Martinelli G, Oehler VG, Papayannidis C, et al. Treatment with PF-04449913, an oral smoothened antagonist, in patients with myeloid malignancies: a phase 1 safety and pharmacokinetics study. Lancet Haematol 2015;2:e339–46. 10.1016/S2352-3026(15)00096-4 26688487

[29] Norsworthy KJ, By K, Subramaniam S, et al. Fda approval summary: glasdegib for newly diagnosed acute myeloid leukemia. Clin Cancer Res 2019;25:6021–5. 10.1158/1078-0432.CCR-19-0365 31064779

[30] Lee JJ, Rothenberg ME, Seeley ES, et al. Control of inflammation by stromal hedgehog pathway activation restrains colitis. Proc Natl Acad Sci U S A 2016;113:E7545–53. 10.1073/pnas.1616447113 27815529 PMC5127312

[31] de la Roche M, Ritter AT, Angus KL, et al. Hedgehog signaling controls T cell killing at the immunological synapse. Science 2013;342:1247–50. 10.1126/science.1244689 24311692 PMC4022134

[32] Smelkinson MG, Guichard A, Teijaro JR, et al. Influenza NS1 directly modulates hedgehog signaling during infection. PLoS Pathog 2017;13:e1006588. 10.1371/journal.ppat.1006588 28837667 PMC5587344

[33] Ye Q, Wang B, Mao J. The pathogenesis and treatment of the "Cytokine Storm" in COVID-19. J Infect 2020;80:607–13. 10.1016/j.jinf.2020.03.037 32283152 PMC7194613

[34] Encinar JA, Menendez JA. Potential Drugs Targeting Early Innate Immune Evasion of SARS-Coronavirus 2 via 2’-O-Methylation of Viral RNA. Viruses 2020;12:525. 10.3390/v12050525 PMC7291090 32397643

[35] Fajgenbaum DC, June CH. Cytokine storm. N Engl J Med 2020;383:2255–73. 10.1056/NEJMra2026131 33264547 PMC7727315

[36] Abidi E, El Nekidy WS, Alefishat E, et al. Tocilizumab and COVID-19: timing of administration and efficacy. Front Pharmacol 2022;13:825749. 10.3389/fphar.2022.825749 35250575 PMC8894855

[37] Tang L, Yin Z, Hu Y, et al. Controlling cytokine storm is vital in COVID-19. Front Immunol 2020;11:570993. 10.3389/fimmu.2020.570993 33329533 PMC7734084

[38] Cavalli G, De Luca G, Campochiaro C, et al. Interleukin-1 blockade with high-dose anakinra in patients with COVID-19, acute respiratory distress syndrome, and hyperinflammation: a retrospective cohort study. Lancet Rheumatol 2020;2:e325–31. 10.1016/S2665-9913(20)30127-2 32501454 PMC7252085

[39] Cavalli G, Larcher A, Tomelleri A, et al. Interleukin-1 and interleukin-6 inhibition compared with standard management in patients with COVID-19 and hyperinflammation: a cohort study. Lancet Rheumatol 2021;3:e253–61. 10.1016/S2665-9913(21)00012-6 33655218 PMC7906668

[40] Crompton T, Outram SV, Hager-Theodorides AL. Sonic hedgehog signalling in T-cell development and activation. Nat Rev Immunol 2007;7:726–35. 10.1038/nri2151 17690714

[41] Li R, Cai L, Ding J, et al. Inhibition of hedgehog signal pathway by cyclopamine attenuates inflammation and articular cartilage damage in rats with adjuvant-induced arthritis. J Pharm Pharmacol 2015;67:963–71. 10.1111/jphp.12379 25645065

[42] Buongusto F, Bernardazzi C, Yoshimoto AN, et al. Disruption of the hedgehog signaling pathway in inflammatory bowel disease fosters chronic intestinal inflammation. Clin Exp Med 2017;17:351–69. 10.1007/s10238-016-0434-1 27655445

[43] Guo Y, Shi G, Wan H, et al. Hedgehog signaling regulates the expression levels of inflammatory mediators in cigarette‑induced airway inflammation. Mol Med Rep 2018;17:8557–63. 10.3892/mmr.2018.8861 29658573

